# Supplementary material for: Adipokines and Endotoxemia Correlate with Hepatic Steatosis in Non-Alcoholic Fatty Liver Disease (NAFLD)
Source: Nutrients. 2020 Mar 5;12(3):699. doi: 10.3390/nu12030699 (PMC7146245; doi:10.3390/nu12030699)
Supplement: Supplementary file 1 [file nutrients-12-00699-s001.pdf]

Supplementary Material: Adipokines and Endotoxemia Correlate with Hepatic Steatosis in Non-Alcoholic Fatty Liver Disease (NAFLD)

Anika Nier [1], Yvonne Huber [2], Christian Labenz [2], Maurice Michel [2], Ina Bergheim [1] and Jörn M. Schattenberg [2,*]

[1] Department of Nutritional Sciences, Molecular Nutritional Science, University of Vienna, Austria
[2] Metabolic Liver Research Program, I. Department of Internal Medicine, University Medical Centre of the Johannes Gutenberg-University, Langenbeckstrasse 1, 55131 Mainz, Germany
Correspondence: joern.schattenberg@unimedizin-mainz.de; Telephone: +49 (0) 6131 17 6074, Telefax: +49 (0) 6131 17 477282

Table S1: Characteristics of Healthy Controls and Patients with Non-alcoholic fatty liver disease (NAFLD)Steatosis

Parameter	Controls	NAFLD
n	14	63
Age	47.4 ± 1.2	49.8 ± 1.6
Sex [m/f]	4/10	33/30
BMI [kg/m2]	23.3 ± 0.7	31.5 ± 0.7 *
Waist circumference [cm]	77.1 ± 2.3	107.9 ± 1.7 *
ALT activity [U/l]	18.4 ± 1.5	70.6 ± 5.6 *
AST activity [U/l]	18.4 ± 1.4	45.8 ± 2.8 *
γ-GT activity [U/l]	18.4 ± 2.3	123.9 ± 22.2 *
Systolic Blood Pressure [mmHg]#	124.4 ± 2.6	140.1 ± 2.4 *
Diastolic Blood Pressure [mmHg]#	82.3 ± 1.7	87.9 ± 1.2 *
Triglycerides [mg/dl]	87.0 ± 16.1	169.3 ± 10.5 *
Total Cholesterol [mg/dl]	195.6 ± 7.2	222.1 ± 5.6 *
HDL-Cholesterol [mg/dl]	72.9 ± 3.4	48.9 ± 1.8 *
LDL-Cholesterol [mg/dl]	107.2 ± 6.7	139.0 ± 5.0 *
Fasting Blood Glucose [mg/dl]#	79.2 ± 2.8	113.8 ± 4.9 *
Fasting Insulin [mU/l]#	4.4 ± 0.4	20.4 ± 5.1 *
HOMA-IR#	0.9 ± 0.1	8.6 ± 3.8 *
Uric Acid [mg/dl]	4.2 ± 0.3	6.2 ± 0.2 *

ALT: alanine aminotransferase, AST: aspartate aminotransferase, γ-GT: γ- glutamyltransferase; Data are shown as total numbers or mean ± standard error of mean (SEM). * $p \leq 0.05$ NAFLD compared to controls. #missing data: blood pressure: n=1, insulin: n=5, fasting glucose: n=1, HOMA: n=6.

Table S2: Nutritional Intake of Healthy Controls and Patients with Steatosis NAFLD

Parameter	Controls	NAFLD
n	12 #	62 #
Total Energy [kcal/]	2194 ± 185	2116 ± 70

Protein [g/d]	82 ± 8	83 ± 3
Fat [g/d]	93 ± 10	93 ± 4
Carbohydrates [g/d]	242 ± 18	227 ± 10
Fiber [g/d]	26 ± 2	19 ± 1 *

Data are shown as total numbers or mean ± standard error of mean (SEM). * $p \leq 0.05$ NAFLD compared to controls. [#] 2 participants in the Control group and 1 participant in the NAFLD group were excluded from the nutritional analysis due to incompliance in dietary assessment.

Table S3: Markers of visceral fat accumulation, intestinal permeability and inflammation in healthy controls and patients with ~~steatosis~~NAFLD.

Parameter	Controls	NAFLD
n	14	38
Leptin [ng/ml]	4.4 ± 0.7	13.1 ± 1.7 *
Adiponectin [ng/ml]	53.2 ± 6.8	26.0 ± 2.9 *
Leptin/Adiponectin	0.08 ± 0.02	0.59 ± 0.08 *
Endotoxin [EU/ml]	0.66 ± 0.03	1.49 ± 0.12 *
LPB [µg/ml]#	25.1 ± 1.9	25.7 ± 1.7
PAI-1 Activity [U/l]	10.63 ± 2.2	47.6 ± 3.4 *
CRP [mg/l]	1.7 ± 0.6	4.8 ± 0.7 *

CRP: c-reactive protein, LBP: lipopolysaccharide binding protein, PAI-1: plasminogen activator inhibitor-1; Data are shown as total numbers or mean ± standard error of mean (SEM). [a] $p \leq 0.05$ compared to controls. #missing data (n=1)
